# Discoloration of Indigo Carmine Using Aqueous Extracts from Vegetables and Vegetable Residues as Enzyme Sources

**DOI:** 10.1155/2013/250305

**Published:** 2013-09-12

**Authors:** A. Solís, F. Perea, M. Solís, N. Manjarrez, H. I. Pérez, J. Cassani

**Affiliations:** ^1^Departamento de Sistemas Biológicos, Universidad Autónoma Metropolitana, Xochimilco, Calzada del Hueso 1100, Colonia Villa Quietud, 09460 Coyoacán, DF, Mexico; ^2^Centro de Investigación en Biotecnología Aplicada, Instituto Politécnico Nacional, Ex-Hacienda San Juan Molino Carretera Estatal Tecuexcomac-Tepetitla Km 1.5, 90700 Tlax, Mexico

## Abstract

Several vegetables and vegetable residues were used as sources of enzymes capable to discolor indigo carmine (IC), completely or partially. Complete discoloration was achieved with aqueous extracts of green pea seeds and peels of green pea, cucumber, and kohlrabi, as well as spring onion leaves. The source of polyphenol oxidase (PPO), pH, time, and aeration is fundamental for the discoloration process catalyzed by PPO. The PPO present in the aqueous extract of green pea seeds was able to degrade 3,000 ppm of IC at a pH of 7.6 and magnetic stirring at 1,800 rpm in about 36 h. In addition, at 1,800 rpm and a pH of 7.6, this extract discolored 300 ppm of IC in 1:40 h; in the presence of 10% NaCl, the discoloration was complete in 5:50 h, whereas it was completed in 4:30 h with 5% NaCl and 2% laundry soap.

## 1. Introduction

The textile industry is one of the most polluting industries, due to the large amounts of water and the great quantity of unused dyes and other chemicals released in wastewaters. Previous studies have shown that textile dyes are toxic to flora and fauna and that their partial degradation products are mutagenic and carcinogenic [1]. Therefore, they present a potential health hazard to all forms of life [2]. Textile industries are the largest consumers of dyes, and it is estimated that 10–15% of the dyes are lost during the dying process and mainly released as sewage [3]. Effluents contaminated with colorants are usually treated by using physical and chemical methods [4]; however, these methods are expensive, lead to the formation of hazardous byproducts, and require high energy input [5]. The use of biological systems for the treatment of these wastewaters has become an attractive alternative [4, 6]. Phytoremediation is valuable to remove or destroy contaminants, because plants can be used to decontaminate soils, industrial sites, brownfields, sediments, and water containing metals and/or organic compounds. It is a low-cost, environmentally friendly technology for the extraction, degradation, or fixation of the contaminants [6, 7]. Oxidoreductases such as polyphenol oxidases (PPOs) and peroxidases have been shown to be effective in the degradation of recalcitrant pollutants from contaminated sites. Peroxidases are enzymes that require hydrogen peroxide or other redox mediators to oxidize a wide variety of inorganic and organic substrates, including dyes; examples of peroxidases used for the discoloration of dyes are those from *Trichosanthes dioica* [8], cauliflower [9], turnip [10, 11], bitter gourd [12], horseradish [13], and chayote [14]. PPOs are a low-cost alternative for the discoloration of dyes because they use free molecular oxygen as oxidant, for example, the PPO from potato and brinjal [15] and banana peel [16]. The purpose of the present study was to evaluate the potential of aqueous extracts of some vegetables and vegetable residues as PPO sources to discolor indigo carmine (IC). The advantages of the selected biological materials are that they are inexpensive and easily accessible; in addition, some of the residues of the selected vegetables such as peels and leaves are considered waste.

## 2. Materials and Methods

### 2.1. Preparation of Aqueous Extracts of Vegetables and Vegetable Residues

The selected vegetables and vegetable residues were obtained from local markets, washed with soap, rinsed with distilled water, immersed in hypochlorite solution (1%) for 5 min, and rinsed again with distilled water. Then, 10 g of the biological material was blended with 20 mL of distilled water using a food processor. The mixture was centrifuged at 10,000 rpm for 10 min. The supernatant was used as enzyme source.

### 2.2. Discoloration of IC Using Aqueous Extracts of Vegetables and Vegetable Residues

IC was added to 3 mL of the aqueous extracts of the vegetables (green pea seed, cucumber, horseradish, and leek) or vegetable residues (peels of green pea, cucumber, lemon, orange, chayote, kohlrabi, and cantaloupe; leaves of turnip, spring onion, and horseradish) to get a final dye concentration of 100 ppm. The mixture was then magnetically stirred (800 rpm) at room temperature until complete discoloration or for a maximum of 36 h. A sample was centrifuged (13,000 rpm for 5 min) to assay the % discoloration of the dye; that is, the absorbance of the supernatant was measured at 610 nm using a UV-Vis spectrophotometer Beckman DU 650 (Beckman Instruments, Inc., USA). The discoloration percentage was calculated as stated in (1):

(1) $$\begin{array}{c} \%\;\text{discoloration}=\frac{A_{o}-A_{f}}{A_{o}}\times 100, \end{array}$$

where *A*_*o*_ = initial absorbance and *A*_*f*_ = final absorbance.

All discoloration experiments were performed in triplicate and average values were determined.

### 2.3. PPO Assay

PPO enzymatic activity of green pea seeds extract was measured using catechol as substrate according to Queiroz et al. [17] with some modifications. A mixture of 1.5 mL of the aqueous extract of green pea seeds (pH 6.32) and 0.5 mL of catechol (0.2 M) in phosphate buffer (pH 6.32, 0.01 M) was incubated at 25°C for 120 min. The blank did not contain enzymatic extract. Absorbance changes were recorded at 420 nm over 3 min, in 30 s intervals, using a spectrophotometer (Beckman DU 650). One unit of PPO activity was defined as the amount of enzyme that caused a change in the absorbance of 0.001 per min.

### 2.4. Protein Analysis

The protein concentration of the extract of green pea seeds was determined by using the dye-binding method of Bradford [18]; bovine serum albumin was used as standard.

### 2.5. PPO Substrate Specificity

The PPO activity of green pea seeds was measured with caffeic acid and pyrogallic acid at concentration of 0.2 mM in phosphate buffer (pH 6.32, 0.1 M). Changes in the absorbance were determined at 420 nm for caffeic acid and at 575 nm for pyrogallic acid. The relative PPO activity was described as the percentage of the activity compared with the activity measured using catechol as substrate.

### 2.6. Effects of pH, Dye Concentration, and Stirring on the Discoloration of IC Using Aqueous Extract of Green Pea Seeds Extract

Aqueous extracts of green pea seeds were made in phosphate buffer (0.1 mM) of various pHs (6.0, 7.0, 8.0, 8.7, and 9.3), prepared according to the procedure described in Section 2.1. Then, IC was added to get a final concentration of 100 ppm, and the mixtures were magnetically stirred at 800 rpm until complete discoloration. Increasing concentrations of IC (50, 100, 200, 500, and 1,000 ppm) were added to the aqueous extract of a pH of 8.7 and the mixtures were stirred at 800 rpm until complete discoloration or for 48 h. The effect of stirring was determined at a pH of 7.0 and 7.6, at 800 and 1,800 rpm, and at an IC concentration of 300 ppm. The reaction was followed until complete discoloration. The % discoloration was calculated using (1).

### 2.7. Effect of NaCl and Soap on the Discoloration of IC Using Aqueous Extract of Green Pea Seeds Extract

To a mixture of IC (300 ppm) and aqueous extract of green pea seeds (pH 7.6), NaCl was added at concentrations of 1, 2, 4, 7, and 10%. The mixtures were stirred at 1,800 rpm until complete discoloration; a sample without salt was used as control. To a mixture of IC (300 ppm) and aqueous extract of green pea seeds (pH 7.6), 5% of NaCl and 2% of commercial laundry soap were added; the mixture was then stirred at 1,800 rpm until complete discoloration.

### 2.8. Detection of the Biotransformation Product

Biotransformation of IC was detected using the aqueous extract of green pea seeds as enzyme source. A mixture of IC (1,000 ppm) and aqueous extract of green pea seeds (pH 7.6) was stirred (1,800 rpm) until complete discoloration. A sample was taken every 1:30 h, centrifuged, and analyzed by high performance liquid chromatography, using an Agilent 1100 Series chromatograph (Agilent Technologies, Inc., Japan) equipped with a diode array detector and a C18-Nucleosil column. Methanol water (50 : 50) was used as mobile phase at a flow rate of 0.5 mL/min. Absorbance was measured at 250 and 610 nm and compared against isatin-5-sulphonic acid (ISA5SA). Samples were analyzed until complete discoloration. 

## 3. Results and Discussion

### 3.1. Discoloration of IC Using Aqueous Vegetable Extracts as Enzyme Sources

**Figure 1:**
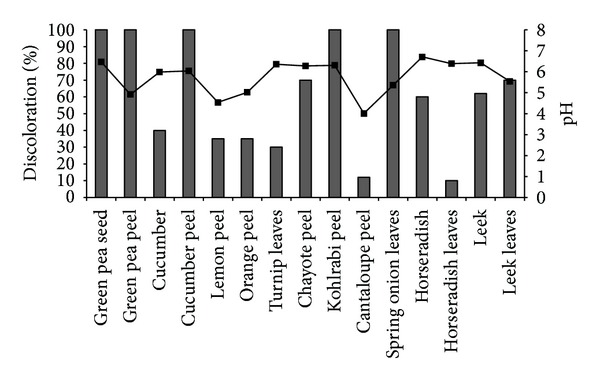
Discoloration of IC (100 ppm) using aqueous extracts of vegetables and vegetable residues as enzyme sources, at 800 rpm. pH –■–.

Several vegetables and vegetable residues were selected to achieve the discoloration of IC. The selected biological materials are easily available in local markets throughout the year. As shown in Figure 1, all tested materials discolored IC to some extent, some completely and others partially. However, green pea seeds are the most interesting material, because their extract was able to discolor 100 ppm of IC completely in about 7 h under stirring at 800 rpm. Almost complete discoloration was achieved with other materials but in longer times, between 24 and 36 h. In addition to edible vegetables such as green peas, cucumber, horseradish, and leek, we thought that it could be interesting to test the parts of plants that are considered waste such as peels and leaves. The vegetable residues that discolored IC completely were the peels from green peas, cucumber, and kohlrabi, as well as the leaves of spring onions; the other peels and horseradish leaves also discolored IC, but to a lesser extent. Some agricultural waste has been used for the discoloration of textile dyes because it involves an adsorption process [19–21]. In the case of the peels and leaves tested in this study, it is not possible that discoloration occurred due to an adsorption process, because we used aqueous extracts as enzyme sources, which means that discoloration was achieved via degradation of the dye. The pH of the reactions was not controlled. They were carried out at the physiological pH of the plants (Figure 1) as a screening to select the aqueous extract with the highest IC discoloration potential. Because the extract of green pea seeds had the highest activity toward the discoloration of IC compared to the other materials, the subsequent experiments were performed using this biological material only.

### 3.2. Enzymatic Analysis

Several monohydroxy-, dihydroxy-, and trihydroxyphenols have been used as substrates to determine PPO activity and specificity, but the most employed substrate for determining PPO activity in plants is catechol [15–17, 22]. Using catechol as substrate, the specific activity of the PPO from the green pea seed extract was determined as 1.288 U min^−1^ protein^−1^. In addition to the fact that PPO oxidizes various phenolic substrates, the highest relative activity was observed in the presence of catechol (100% of the relative activity), followed by caffeic acid (48.23%) and pyrogallic acid (43.91%).

### 3.3. Effect of the pH, Dye Concentration, and Stirring on the Discoloration of IC Using an Aqueous Extract of Green Pea Seeds

**Figure 2:**
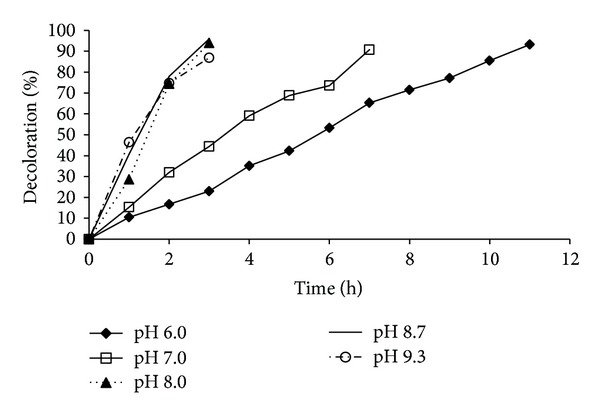
Effect of the pH on discoloration of IC (100 ppm) using green pea seed extract, under stirring at 800 rpm.

Enzymes have a characteristic pH at which they show maximum activity. To determine the pH at which significant IC discoloration occurs, buffers of various pHs (6.0, 7.0, 8.0, 8.7, and 9.3) were used. The % discoloration of IC (100 ppm, at 800 rpm) at different pHs over time is shown in Figure 2. An acidic pH (6.0) was not favorable for the discoloration of the dye. A basic pH was more favorable for the enzymatic activity of the green pea seed extract. In the first 2 h, the degree of discoloration was similar at pH 8.0, 8.7, and 9.3, but within the next hour, 93% discoloration was achieved at pH 8 and 8.7, whereas only 84% was achieved at pH 9.3. Some authors have reported that other PPOs are more active at an acidic pH; for example, maximum discoloration with PPOs from potato (*Solanum tuberosum*) and brinjal (*Solanum melongena*) was observed at a pH of 3.0 [15], whereas the PPO from banana peel was more active at a pH of 7.0 [16].

Table 1 shows the % discoloration of IC at different dye concentrations, using the aqueous extracts of green pea seeds at a pH of 8.7 and magnetic stirring at 800 rpm. Under this condition, 200 ppm of IC was almost completely discolored in 4:30 h; however, in the presence of 1,000 ppm of IC, only 48.85% discoloration was achieved in 48 h. The next experiment was performed to find out if the enzymatic extract is stable at a pH of 8.7. The extract was stirred at 22°C and a pH of 8.7 for 5 h. Then, IC was added to the extract to get a final concentration of 100 ppm. Stirring was continued for an additional 24 h, but the % discoloration was negligible. It is possible that the maximum activity of the extract at a pH of 8.7 lasts only for a few hours, which could be explained by some kind of inactivation that is taking place at a higher pH. 

In addition to the source of the enzyme, time, pH, and the concentration of the dye, we observed that aeration by stirring is also very important. The discoloration of IC (300 ppm) was studied at pHs of 7 and 7.6 and the reaction was carried out under stirring at 800 and 1,800 rpm until complete discoloration. The results are shown in Table 2. A high stirring rate and, consequently, high aeration are favorable for the reaction. The time required to discolor the dye was lowered considerably under stirring at 1,800 rpm compared with 800 rpm, and this could be because PPO requires free molecular oxygen as oxidant [22, 23]. The PPO activity of the green pea seeds was more efficient at a pH of 7.6. For example, at high-speed stirring (1,800 rpm), discoloration of 3,000 ppm of IC was achieved within 36 h with the aqueous extract of green pea seeds (pH 7.6). 

Discoloration of 3,000 ppm of IC with green pea seed extract was achieved within 36 h at a pH of 7.6 and stirring at 1,800 rpm. This is a very interesting improvement, compared with other studies on the discoloration of IC. For example, Neelamegam et al. [24] achieved 90% discoloration of IC (100 ppm) in 6 days using *Pleurotus ostreatus.* Ramya et al. [25] used a liquid culture of *Paenibacillus larvae *with solutions containing 100 ppm IC and achieved 100% of discoloration in 8 h. Podgornik et al. [26] reported the biodegradation of 30 ppm of IC in 2 h using isozymes of lignin peroxidase and manganese peroxidase obtained from *Phanerochaete chrysosporium*; an IC solution of 23.31 ppm was completely adsorbed on chitosan in 3.3 h [27].

### 3.4. Effect of NaCl and Soap on the Discoloration of IC Using an Aqueous Extract of Green Pea Seeds

In the textile finishing process, it is very common to use NaCl and soap, in addition to dyes. Thus, it was important to determine if the enzymes present in the green pea seed extract are able to degrade IC under such conditions, and if they are stable in the presence of NaCl and soap. The experiment was done with a solution of IC (300 ppm) and increasing quantities of NaCl, at a pH of 7.6 and stirring at 1,800 rpm. The control sample did not contain NaCl. From the results shown in Table 3, it can be perceived that the enzymes present in the aqueous extract of green pea seeds are able to discolor the dye completely, even in the presence of 10% NaCl. The biocatalytic activity of green pea seeds can be compared to that of halotolerant and halophilic microorganisms that are capable to discolor azo dyes in the presence of 4 to 15% of NaCl [28]. There are some reports about the reduction of the PPO activity in the presence of NaCl [29].

Complete discoloration of IC (300 ppm) was achieved in 4:30 h using an aqueous extract of green pea seeds in the presence of 5% NaCl and 2% commercial laundry soap (pH 7.6, 1,800 rpm). Thus, the enzymes present in the extract are active under the tested reaction conditions. This is a very important finding, because, in addition to colorants, high quantities of NaCl and soap are used in the textile industry. Therefore, the enzymes present in the extract may be useful in the wastewater treatment.

### 3.5. Biotransformation of IC

**Figure 3:**
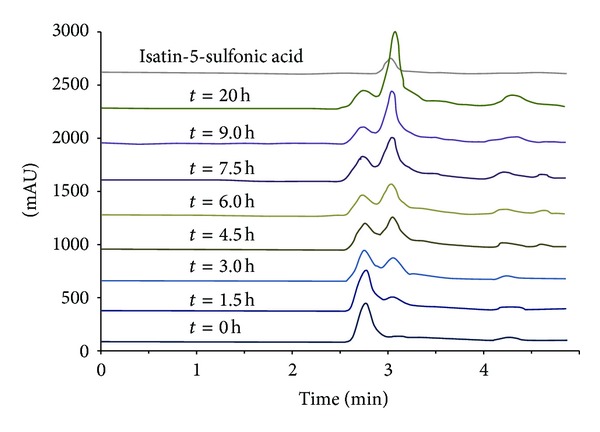
Chromatograms for the discoloration of IC using aqueous extracts of green pea seeds as PPO source, *λ* = 250 nm.

The discoloration of IC (1,000 ppm) with the aqueous extract of green pea seeds was measured by high performance liquid chromatography for 20 h and compared with ISA5SA, the previously reported IC degradation product [30, 31]. The results are shown in Figure 3. The peak corresponding to IC was detected at 610 nm with a retention time of 2.717 min; the peak of ISA5SA was detected at 250 nm and had a retention time of 2.992 min. At the 0 h time point, peaks corresponding to IC and some compounds present in the extract could be detected in the reaction mixture containing IC and the aqueous extract of green pea seeds. The principal peak corresponding to a compound from green pea seeds was detected at 2.645 min. Every 1.5 h, a sample was analyzed by high performance liquid chromatography. We observed that the peak at 2.992 min, corresponding to ISA5SA, was growing constantly, and the IC concentration diminished with time (data not shown). Thus, we can assume that IC is being degraded to ISA5SA by the action of a PPO in the extract of green pea seeds.

## 4. Conclusions 

PPOs from vegetables and vegetable residues tested in this study are able to discolor IC without requiring any redox mediator. Both materials are inexpensive and easily accessible. The potential use of waste such as vegetable peels is very attractive because it can help to reduce the pollution due to food waste. In addition, the waste is a source of enzymes that are useful in bioremediation treatments. The PPO from green pea seeds discolored IC even in the presence of NaCl and laundry soap, which is advantageous because those products are commonly employed in the dyeing processes in the textile industry. The results presented in this work are interesting because with this simple system that uses crude enzymatic extracts from inexpensive materials, that is, vegetables, we obtained similar or even better results than those described in other reports in which different or even more sophisticated and expensive processes were used. 

## Figures and Tables

**Table 1:** Effect of the concentration of IC on the discoloration using green pea seed extracts at a pH of 8.7 and stirring at 800 rpm.

IC (ppm)	Time (h)	% discoloration
50	2:30	97.74
100	3:00	95.99
200	4:30	98.41
500	24:00	84.05
1000	48:00	48.85

**Table 2:** Effect of stirring and pH on the time to achieve complete discoloration of IC, using green pea seed extract as enzyme source.

pH	IC (ppm)	Agitation (rpm)	Time (h) to complete IC discoloration
7.0	300	1800	3:20
7.0	300	800	7:00
7.6	300	1800	1:30
7.6	300	800	4.30
7.6	3000	1800	36:00

**Table 3:** Discoloration of IC (300 ppm) with an aqueous extract of green pea seeds in the presence of NaCl (pH 7.6 and 1,800 rpm).

%NaCl	1	2	4	7	10	Control
Time (h)	2:15	2:30	3:10	3:50	5:50	1:30
